# A Cohort Historical Analysis of the Relationship between Thyroid Hormone Malady and Alpha-Human Herpesvirus Activation

**DOI:** 10.4172/2157-7536.1000133

**Published:** 2014-06-04

**Authors:** Shao-Hsuan Hsia, Victor Hsia S

**Affiliations:** 1 Department of Pediatrics, Division of Pediatric Critical Care Medicine, Chang Gung Memorial Hospital, College of Medicine, Chang-Gung University, Kweishan, Taiwan; 2 School of Pharmacy, University of Maryland Eastern Shore, Princess Anne, MD 21853, USA

**Keywords:** αHHV, HSV-1, Thyroid Hormone, Retrospective study, Viral reactivation, Risk ratio, Odds ratio, Medical claims, ICD-9 diagnostic codes

## Abstract

**Background:**

A number of physiological factors have been suggested to participate in the alpha- Human Herpesvirus (αHHV) reactivation, such as hormonal aberration. Thyroid hormone (TH) was shown to play a suppressive role in Herpes Simplex Virus Type-1 (HSV-1) gene expression and replication in cell culture and animal models. We hypothesize that reactivation of αHHV in humans may be due to, at least in part, by TH status.

**Methods:**

Prior to implementing a full-scale population-based prospective inquiry into this hypothesis, a pilot study using a medical claims data base and a case-controlled, retrospective cohort investigation was conducted to develop a hypothetical link between TH complication and αHHV reactivation. Using diagnostic codes for treating thyroid disorders and αHHV infections as proxies for biologic/clinic outcomes, we queried a large, comprehensive hospital data base to construct two patient cohorts: Cohort 1 was comprised of patients receiving TH diagnoses over a twelve-year period, and Cohort 2 was composed of patients not receiving TH diagnoses during this period. Diagnoses of αHHV were recorded for each cohort and the difference in the frequency was examined for statistical significance. Demographic analyses such as age, gender, etc were also performed.

**Results:**

Using 2×2 contingency table analyses and Statistical Analysis Software (SAS), an Odds Ratio (OR) of 2.83 was observed for the total population of 21 years old and above with a chi-square of 61.55 and p < 0.001, confirming that a severe significant difference was found between these two cohorts. This result suggested that patients with αHHV diagnosis have higher chances to have TH disorders. Additional investigation revealed that female were at higher/significant probability to have both TH and αHHV diagnosis, indicating a link of αHHV reactivation to a complex hormonal profile difference between genders. Our observation indicated that female patients of 21 years of age and above exhibited a very high incidence (OR of 3.40, p < 0.001) compared to the male groups (OR of 1.91, p < 0.05), indicating the possibility that hormonal alteration in females maybe transient but robust and can lead to αHHV reactivation more often than the males.

**Conclusion:**

These results indicated that TH dysfunction may have implication in αHHV pathogenesis and females exhibited much higher probability to suffer αHHV reactivation due to TH disruption. Although the results from this pilot study have limitations and require additional controlled clinical examination such as more detailed patient records, lab data, therapeutic outcome, etc, it provides a tool to assess the effects of hormone imbalance on virus reactivation by retrospective analyses using existing large scale data base.

## Introduction

Thyroid hormone (TH or T3) is known to have important regulatory functions for normal biological processes. TH produces its physiological effects primarily through its nuclear receptor (TR), which is a transcriptional factor that controls gene expression and the net outcome, is determined by TH level [1]. Hormone imbalances have been suggested to affect viral pathogenesis [2]. Latest literatures proposed that TH participated in the regulation of HSV-1 gene silencing and replication and may have influence during viral latency/ reactivation [3,4].

HSV-1 belongs to the superfamily of alpha Human Herpesvirus (αHHV) and represents one of the most widespread infections [5]. Besides HSV-1, HSV-2 and Varicella Zoster Virus (VZV) are also grouped in the αHHV category sharing close similarity of their genomes [6–9]. These viruses are widespread in US population, evidenced by the sero-prevalence rates of 57.7% [10]. These viruses have a complex infection pattern. Following the lytic assault of epithelial cells, the virus may enter sensory neurons of trigeminal ganglia and establish latency, in which the virus remained dormant without gene expression and replication. The reactivation happened temporarily at different sites connected to the trigeminal nerves such as the mucosa, lips, eyes, or perioral area of the face causing minor lesions such as fever blisters [11]. Encephalitis caused by HSV-1 reactivation accounted for approximately 10% of all viral encephalitis [12]. The molecular mechanisms leading to αHHV reactivation remain ambiguous [13].

Literatures and clinical studies reported that episodes of stress, facial injury, etc. that triggered reactivation of αHHV also showed evidences of decreased level of TH levels [2]. The working hypothesis is that αHHV latency and reactivation may be affected, at least in part, by the TH status. In another word, transient hypothyroidism may alleviate TH-mediated suppression of viral replication and promote viral gene expression and reactivation. However, there is no controlled clinical evidence in human subjects supporting this hypothesis.

## Materials and Methods

### Study design

To support the working hypothesis from pre-clinical standpoints, a case-control, retrospective investigation querying a hospitalization data base from a large research/teaching hospital was performed using diagnoses codes as a measure of disease status. This type of research approach is considered as a prerequisite step in the further development of prospective clinical investigation to test the hypothesis that anomalous TH level is associated with reactivation of the αHHV.

### Study populations

**Figure 1:**
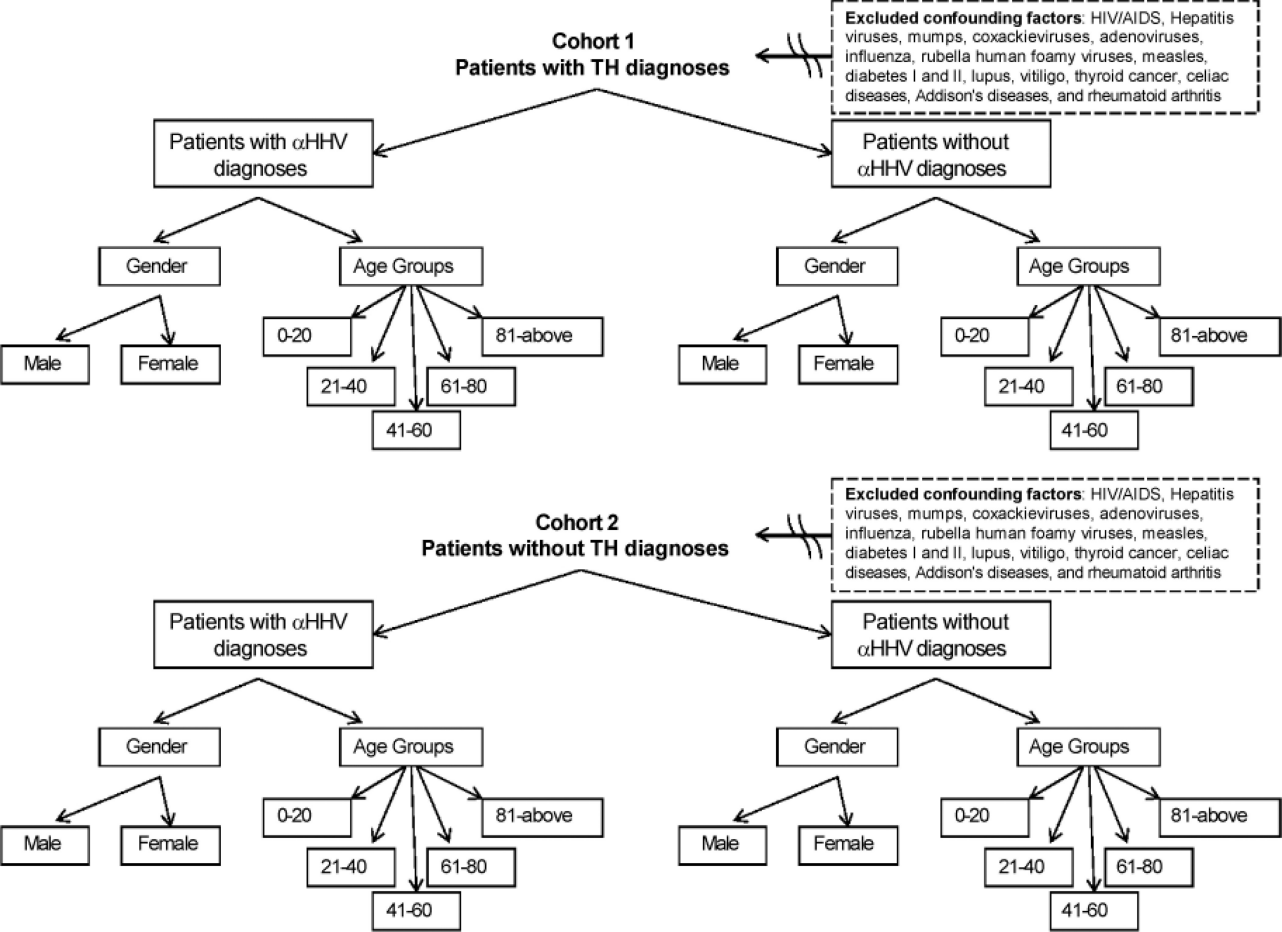
Experimental design of the cohort studies. ICD9 diagnosis codes were collected from 2001–2012 and divided into two groups based on the diagnoses of their TH level. After categorized into two cohorts with or without TH diagnoses, each group was further separated into different units based on their gender and age. To ensure the data validity, confounding factors were cleared.

The data of patients who were hospitalized within a twelve-year period (2001–2012) were obtained from Chang Gung Memorial Hospital, a large research/teaching comprehensive hospital in Taiwan. The ICD-9 codes for Disorders of Thyroid Gland (240.xx to 246.xx) and αHHV infections (VZV: 053.xx and HSV: 054.xx) were collected and analyzed. Demographic data such as years, genders, age groups, etc were assessed to comprehend the features of this population. Of all the patients receiving examination within this twelve-year period, the following specific diagnosis data were categorized: 1) total number of patients for all diagnoses within the time period; 2) number of patients without diagnoses of αHHV and thyroid dysfunction; 3) number of patients with diagnoses for thyroid dysfunction; 4) number of patients with diagnoses for thyroid malady and no diagnoses of αHHV; 5) number of patients with diagnoses of αHHV; 6) number of patients receiving diagnoses of αHHV without diagnoses for thyroid dysfunction; and 7) number of patients receiving diagnoses of both αHHV AND thyroid disorders. This analysis was also performed on different genders and age groups to compare the effects of putative sex differences. The strategy was summarized in Figure 1.

### Eligibility criteria

Confounding factors were removed to ensure the eligibility and authenticity of the results. Factors excluded are HIV/AIDS, Hepatitis viruses, mumps, coxackieviruses, adenoviruses, influenza, rubella human foamy viruses, measles, diabetes I and II, lupus, vitiligo, thyroid cancer, celiac diseases, Addison's diseases, and rheumatoid arthritis. Specific codes were used for the purposes of exclusions (Figure 1).

### Statistical analysis

To examine the hypothetical risk of developing αHHV disease if encountered TH disorders, we performed historic cohort statistical analyses (2×2 contingency table analyses and Statistical Analysis Software (SAS)) to measure an odds ratio or risk ratio for assessment of relative risk. In another word, higher relative risk number represents higher probability of developing a αHHV disease (risk) related to TH disorder (condition). For example, If a virus outbreak is linked to thyroid dysfunction, then a significant difference in the diagnoses should be obvious between the cohorts, evidenced by a calculated Odds Ratio (OR) of >1 and a significant p-value would indicate a correlation between thyroid abnormality and viral infection, as described by the alternative measure of diagnoses. On the contrary, null hypothesis would be presented as no difference between these two cohorts, indicating that thyroid malady is not linked to activation of virus. To analyze these two groups of subjects, each cohort was sorted according to the absence (−) or presence (+) of the correspondent condition, this statistical software then calculated standard measures for Rates, Risk Ratio, Odds, Odds Ratio, etc. Risk Ratio was calculated as Rate (group 1)/Rate (group 2); rate reflects the proportion in group with condition present. Odds Ratio was measured as Odds (group 1)/ Odds (group 2), as Odds (group 1) = present (group 1)/absent (group 1) and Odds (group 2) = present (group 2)/absent (group 2).

## Results

### Characteristics of the Patients

A total of 1,516,372 patients (male: 759,904; female: 756,468) were hospitalized from 2001 to 2012 and the ICD-9 codes were obtained anonymously.

**Figure 2:**
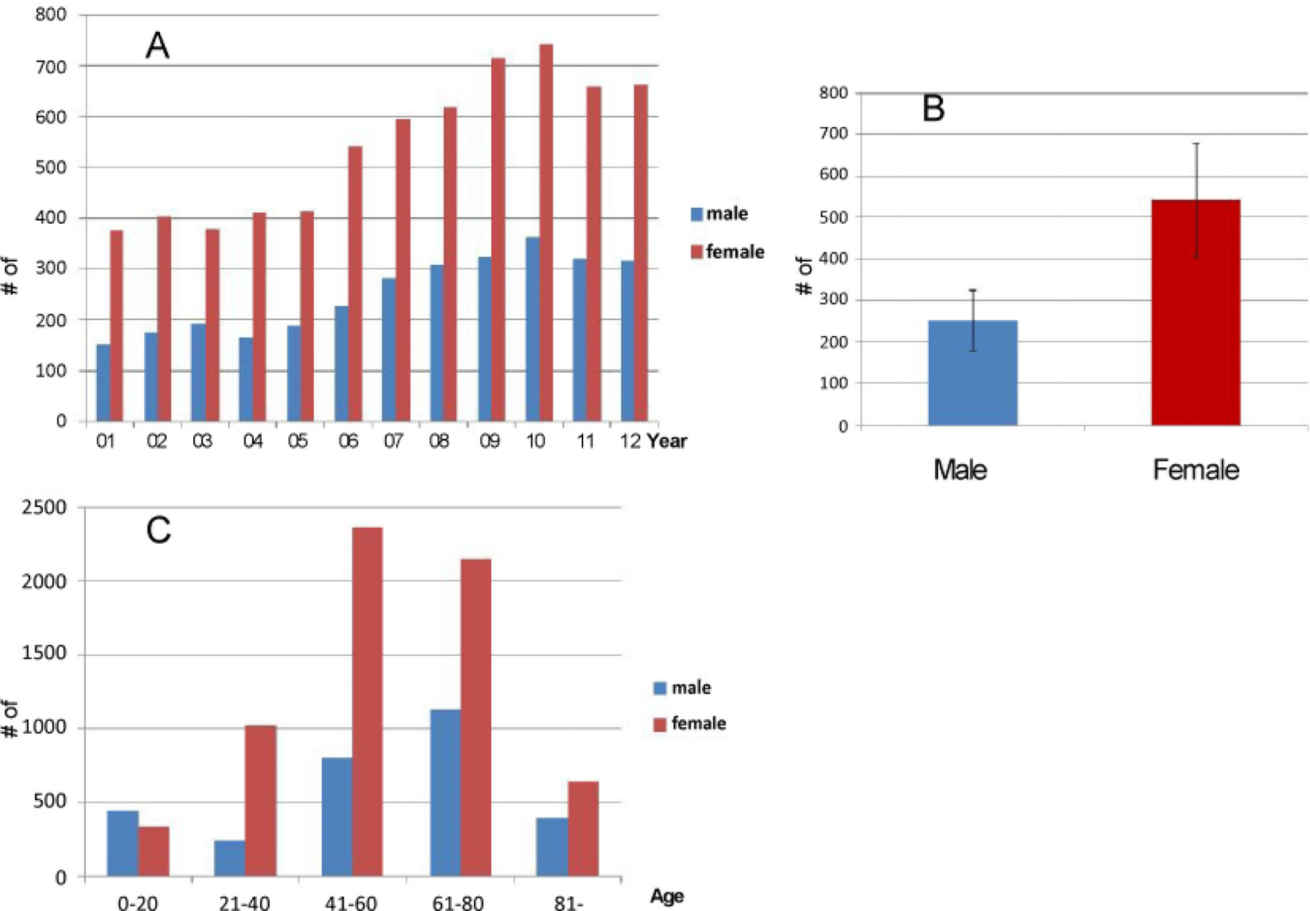
Analyses of patients with TH diagnoses. A. TH diagnoses between 2001–2012 B. Gender difference of patients suffering TH dysfunction from 2001–2012. The p value is <0.05. C. TH diagnoses in different age groups

**Figure 3:**
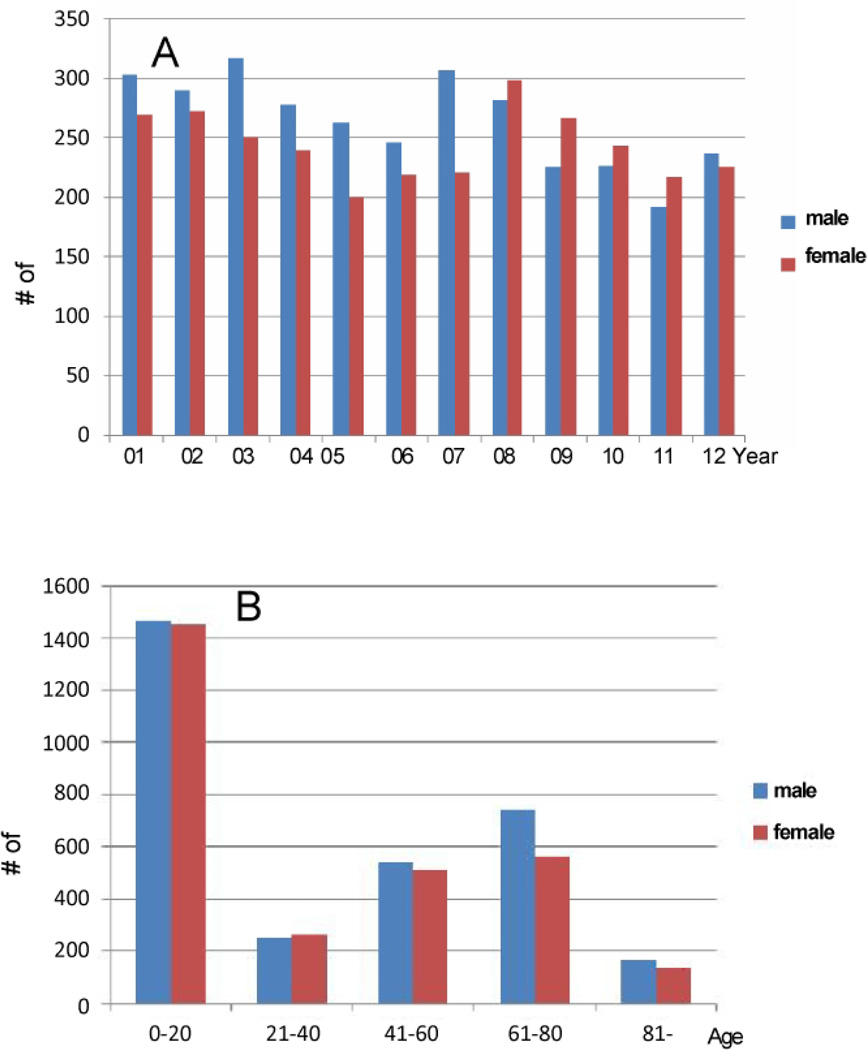
Analyses of patients with αHHV diagnoses. A. αHHV diagnoses between 2001–2012 B. αHHV diagnoses in different age groups

Patients (both male and female) diagnosed with TH disorders increased gradually throughout the years and peaked in 2009–2010 and remained high in 2011 and 2012 (Figure 2A). Based on the existing records from the database (2001–2012), females had twice as much TH disorders than the males (Figure 2B). In addition, there was no gender difference regarding TH abnormality before twenty years of age (Figure 2C). However, TH dysfunction became a prominent issue for females over 21 years old compared to the male counterpart, especially for those who were 21–80 years of age (Figure 2C). As for the αHHV diagnoses, young patients under 20 in both genders had a very high numbers of hospitalization record (Figure 3A and 3B). There was no gender difference in adult αHHV diagnoses but the age group of 61–80 exhibited higher cases of virus-related hospitalization (Figure 3B).

### Association of TH dysfunction and αHHV reactivation

**Figure 4:**
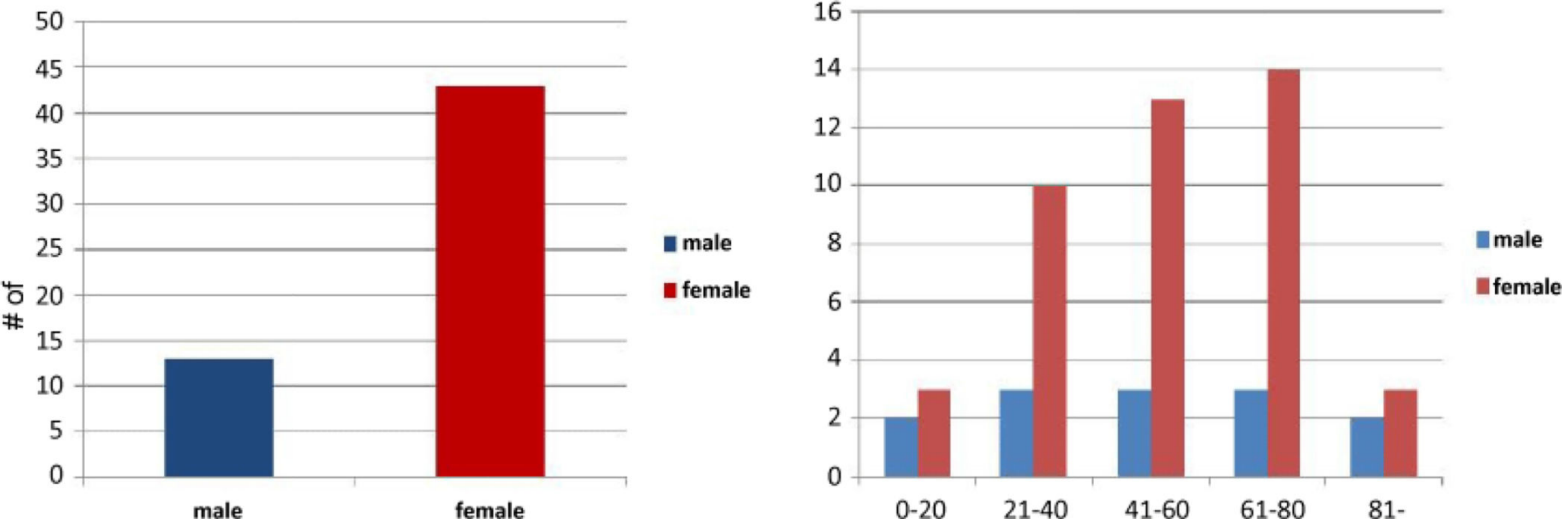
Analyses of patients with both TH and αHHV diagnoses. A. αHHV+TH+ diagnoses between genders B. αHHV+TH+ diagnoses among age groups

After the screening of confounding factors and removal of unqualified diagnoses, the computer analyses showed that 56 patients possessed both TH and αHHV diagnoses codes, 13 males and 43 females, during 2001–2012 (Figure 4A). Additional investigations analyzing among age groups indicated that female patients between 21–80 years of age exhibited much higher diagnoses than the same age group of males (Figure 4B). This finding probably resulted, at least in part, from the fact that more females in these age groups were diagnosed with TH disorders than the males (Figure 2B).

To address the relative risk of αHHV infection related to a condition of TH dysfunction, OR/RR were measured for assessment. The results showed that the OR/RR was approximately 1.46 for total hospitalized population of all ages between 2001and 2012 with p value less than 0.005 (Table 1A), indicating that patients suffering αHHV infections exhibited increased chance of having TH disorders.

Further studies revealed that female patients possessed slightly increased relative risk by showing an OR of 1.72 with p value less than 0.005 (Table 1B).

Male patients, however, showed no significant difference between patients suffering TH disorder or not (Table 1C). Together these observations suggested that TH dysfunction-related αHHV reactivation were more obvious in females than males.

Our additional analyses displayed the differences of patients diagnosed with αHHV and TH disorders among age groups (Figure 4B). Thus we performed statistical analyses excluding patients under 20 years of age of both genders and focusing on patients of 21 years old and above. The results demonstrated that it is significant with an OR of 2.83 and p value less than 0.001 (Table 2A).

If concentrated on females over 21 years of age the statistics is even more significant with OR of 3.40 and p value less than 0.0001 (Table 2B), compared to males (OR: 1.91) with p value less than 0.05 (Table 2C). Collectively these data indicated that adult females exhibited very high probability of suffering TH malady-associated αHHV infections than the male counterpart.

## Discussion

### Rationales

The relationship between αHHV reactivation and TH dysfunction were not discussed until 2010 when publications showed that TH can suppress the replication and gene expression of HSV-1, a αHHV, in cultured neuronal cells [3,4]. Bioinformatics revealed several putative TH responsive elements within the HSV-1 regulatory sequences and were identified to produce regulatory effects by molecular biology studies [3,4]. There are rationales to suggest TH level can affect αHHV reactivation and latency. For example, both TH and its nuclear receptors are available in ganglia neurons where HSV-1 established latency [14,15] and can modulate neuronal survival, differentiation, maturation [16], raising the possibility of regulating the HSV-1 latency/reactivation. Recent evidences indicated that TH participated in controlling neurite outgrowth and axonal elongation/transport [17], both are critical for HSV-1 latency, via nerve growth factor (NGF)-TrkA-PI3 Kinase signaling pathway using a unique non-genomic mechanism [18]. It is likely that TH enhances NGF-mediated PI3-Kinase cascade to promote HSV-1 latency and transient low TH may instead increase the chance of reactivation. However, direct clinical or epidemiological studies regarding the effect of TH on HSV-1/VZV reactivation, to our knowledge, are not available at this point.

### Clinical significance

Many scenarios that reduce TH levels can trigger HSV-1 reactivation [2]. For instance, a clinical study indicated that a patient with myxedema coma under corticosteroid regime suffered severe herpes simplex encephalitis diagnosed with extremely low thyroxine level less than 5.2 nmol/L (normal range 12–30 nmol/L) [19]. Furthermore, it is well documented that corticosteroid changes have been shown to induce HSV-1 reactivation in animal studies [20,21]. These observations prompted us to perform a large-scale population-based cohort examination to examine this hypothesis using existing hospitalized data.

### Disparity of genders and ages

Our analyses confirmed the general opinion that females exhibited more TH dysfunction than males (Figure 2A and 2B). The data also showed that more TH dysfunctions were diagnosed while people age with peak observed at 41–60 years of age (Figure 2C). Although we do not observe the gender/age difference in αHHV diagnoses, the results demonstrated that females had significantly higher odds ratio than males when we linked the relationships of TH malady and αHHV infections, particularly between 21–80 years of age (Figure 4 and Table 2). It is likely due to the complex profiles of hormonal regulation in females or other influence from female dominant hormones. The exact mechanisms are yet clear.

During an independent study, our collaborators queried a regional hospital database in US using similar strategy. The preliminary results showed that the OR of HSV-1 patient’s experienced TH dysfunction for total population is 8-time higher (unpublished data). It is likely due to under- diagnoses. However, we do not exclude the possibilities of health disparity and/or cultural/environmental factors.

Thyroid hormone malady/autoimmunity affected adults as well as children significantly [22,23]. The correlation is not significant in young patients especially in children. However, the correlation may be underestimated because many children with primary hypothyroidism are subclinical or asymptomatic and remain under-diagnosed [24–26].

### Advantages and Disadvantages

This kind of retrospective design has some marked advantages over prospective studies since the historical investigation characteristically involve shorter period of time to complete since patients suffering disorders have already been identified and diagnosed. This fact would be generally beneficial since it is less expensive to carry on the studies and the resources should be focused on the collection and analyses of data only.

Retrospective studies have several drawbacks such as the bias of data selection and statistical difficulties, etc. that may influence the validity. In addition, the chronological connection of exposures and outcomes are usually challenging for assessment but an accurate record-keeping may be able to resolve the issues.

## Conclusion

The present study suggested that TH level may play a critical role in αHHV reactivation and TH levels can become a standard biomarker for recurrent αHHV diagnoses. Furthermore, physicians can be advised of the future relative risk of αHHV occurrence of their patients and appropriate medical TH treatment may be considered while treating patients of HSV-1, HSV-2, and VZV. The results of the pilot study provided justification to the hypothetical link between anomalous TH level and reactivation of the αHHV in humans. It is noted that the number of patients possessed both TH and alpha-HHV diagnoses codes is tremendously low compared to the total number of patients in the database. It is likely due to the number of our confounding factors since a lot of cases were removed to avoid complication from other diseases. Consequently, more investigations from other databases are necessary and additional population- based prospective investigation is of importance to assess more accurate nature of the connection.

## Figures and Tables

**Table 1A:** Statistical analyses of odds ratio of all age groups; Of total population, p value < 0.005

		αHHV		
		**−**	**+**	**Total**
**TH**	**+ (group 1)**	9484	56	9540
	**− (group 2)**	1500799	6033	1506832
	**Total**	1510283	6089	1516372
			**.95 Confidence Intervals**	
		**Observed**	**Lower limit**	**Upper limit**
	**Risk Ratio**	1.4661	1.1272	1.906
	**Odds Ratio**	1.4689	1.1282	1.9125
		**Chi-Square**		
	**Phi**	**Yates**	**Pearson**	
		7.8	8.26	
	**p**	0.00523	0.004053	

**Table 1B:** Statistical analyses of odds ratio of all age groups; Of all females, p value < 0.001

		αHHV		
		**−**	**+**	**Total**
**TH**	**+ (group 1)**	6484	43	6527
	**− (group 2)**	747065	2876	749941
	**Total**	753549	2919	756468
			**.95 Confidence Intervals**	
		**Observed**	**Lower limit**	**Upper limit**
	**Risk Ratio**	1.7179	1.2725	2.3192
	**Odds Ratio**	1.7226	1.2735	2.3302
		**Chi-Square**		
	**Phi**	**Yates**	**Pearson**	
		12.05	12.76	
	**p**	0.000518	0.000354	

**Table 1C:** Statistical analyses of odds ratio of all age groups; Of all males, p value is 0.88

		αHHV		
		**−**	**+**	**Total**
**TH**	**+ (group 1)**	2999	13	3012
	**− (group 2)**	753736	3156	756892
	**Total**	756735	3169	759904
			**.95 Confidence Intervals**	
		**Observed**	**Lower limit**	**Upper limit**
	**Risk Ratio**	1.03	0.6	1.78
	**Odds Ratio**	1.03	0.6	1.78
		**Chi-Square**		
	**Phi**	**Yates**	**Pearson**	
		0	0.02	
	**p**	1	0.88	

**Table 2A:** Statistical analyses of odds ratio over 21 years of age; Of total population, p value < 0.001

		αHHV		
		**−**	**+**	**Total**
**TH**	**+ (group 1)**	8787	51	8838
	**− (group 2)**	1504000	3200	1507200
	**Total**	1512787	3251	1516038
			**.95 Confidence Intervals**	
		**Observed**	**Lower limit**	**Upper limit**
	**Risk Ratio**	2.82	2.15	3.70
	**Odds Ratio**	2.83	2.16	3.72
		**Chi-Square**		
	**Phi**	**Yates**	**Pearson**	
		59.75	61.55	
	**p**	<.0001	<.0001	

**Table 2B:** Statistical analyses of odds ratio over 21 years of age; Of females, p value < 0.001

		αHHV		
		**−**	**+**	**Total**
**TH**	**+ (group 1)**	1434	40	1474
	**− (group 2)**	750036	6142	751678
	**Total**	751470	6182	757652
			**.95 Confidence Intervals**	
		**Observed**	**Lower limit**	**Upper limit**
	**Risk Ratio**	3.34	2.45	4.54
	**Odds Ratio**	3.40	2.48	4.67
		**Chi-Square**		
	**Phi**	**Yates**	**Pearson**	
		63.4	65.72	
	**p**	<.0001	<.0001	

**Table 2C:** Statistical analyses of odds ratio over 21 years of age; Of males, p value < 0.05

		αHHV		
		**−**	**+**	**Total**
**TH**	**+ (group 1)**	1691	11	1702
	**− (group 2)**	753895	2559	756454
	**Total**	755586	2570	758156
			**.95 Confidence Intervals**	
		**Observed**	**Lower limit**	**Upper limit**
	**Risk Ratio**	1.91	1.05	3.44
	**Odds Ratio**	1.91	1.05	3.47
		**Chi-Square**		
	**Phi**	**Yates**	**Pearson**	
		3.9	4.77	
	**p**	0.048	0.028	
